# A Novel Inflammation-Based Stage (I Stage) Predicts Overall Survival of Patients with Nasopharyngeal Carcinoma

**DOI:** 10.3390/ijms17111900

**Published:** 2016-11-15

**Authors:** Jian-Pei Li, Shu-Lin Chen, Xiao-Min Liu, Xia He, Shan Xing, Yi-Jun Liu, Yue-Hao Lin, Wan-Li Liu

**Affiliations:** State Key Laboratory of Oncology in South China, Collaborative Innovation Center for Cancer Medicine, Department of Clinical Laboratory Medicine, Sun Yat-Sen University Cancer Center, Guangzhou 510060, China; lijp@sysucc.org.cn (J.-P.L.); chenshl@sysucc.org.cn (S.-L.C.); liuxm@sysucc.org.cn (X.-M.L.); hexia@sysucc.org.cn (X.H.); xingshan@sysucc.org.cn (S.X.); liuyij@sysucc.org.cn (Y.-J.L.)

**Keywords:** nasopharyngeal carcinoma, inflammation-based stage, survival, prognosis

## Abstract

Recent studies have indicated that inflammation-based prognostic scores, such as the Glasgow Prognostic Score (GPS), modified GPS (mGPS) and C-reactive protein/Albumin (CRP/Alb) ratio, platelet–lymphocyte ratio (PLR), and neutrophil–lymphocyte ratio (NLR), have been reported to have prognostic value in patients with many types of cancer, including nasopharyngeal carcinoma (NPC). In this study, we proposed a novel inflammation-based stage, named I stage, for patients with NPC. A retrospective study of 409 newly-diagnosed cases of NPC was conducted. The prognostic factors (GPS, mGPS, CRP/Alb ratios, PLR, and NLR) were evaluated using univariate and multivariate analyses. Then, according to the results of the multivariate analyses, we proposed a I stage combination of independent risk factors (CRP/Alb ratio and PLR). The I stage was calculated as follows: patients with high levels of CRP/Alb ratio (>0.03) and PLR (>146.2) were defined as I2; patients with one or no abnormal values were defined as I1 or I0, respectively. The relationships between the I stage and clinicopathological variables and overall survival (OS) were evaluated. In addition, the discriminatory ability of the I stage with other inflammation-based prognostic scores was assessed using the AUCs (areas under the curves) analyzed by receiver operating characteristics (ROC) curves. The *p* value of <0.05 was considered to be significant. A total of 409 patients with NPC were enrolled in this study. Multivariate analyses revealed that only the CRP/Alb ratio (Hazard ratio (HR) = 2.093; 95% Confidence interval (CI): 1.222–3.587; *p* = 0.007) and PLR (HR: 2.003; 95% CI: 1.177–3.410; *p* = 0.010) were independent prognostic factors in patients with NPC. The five-year overall survival rates for patients with I0, I1, and I2 were 92.1% ± 2.9%, 83.3% ± 2.6%, and 63.1% ± 4.6%, respectively (*p* < 0.001). The I stage had a higher area under the curve value (0.670) compared with other systemic inflammation-based prognostic scores (*p* < 0.001). The I stage is a novel and useful predictive factor for OS in patients with NPC.

## 1. Introduction

Nasopharyngeal carcinoma (NPC) is a common squamous-cell carcinoma, with an extremely skewed distribution across the world. Being endemic in Southeast Asia, the incidence of NPC peaks in the age range of 40 to 50 years, which has caused great problems in society. The adoption of radiotherapy with chemotherapy or without chemotherapy as the primary treatment modality for patients with NPC has led to five-year survival rates now exceeding 75% [1]. Epstein Bar virus (EBV) infection, genetic and environmental factors, lifestyle, and smoking are closely related to NPC [2,3,4]. Currently, the optimal method of assessing the prognosis of patients with NPC is based primarily on the Union Internationale Contrele Cancer/American Joint Cancer Committee (UICC/AJCC) tumor-node-metastasis (TNM) staging system, which was the most widely used set of parameters to formulate rational treatment strategies and to predict clinical outcomes. Nevertheless, with the current TNM staging system, without considering the biological variability of the tumor itself, there were large variations in clinical outcomes that can be found in NPC patients with the same stage and similar treatment regimens [5]. Thus, the prognostic value of the TNM staging system, in terms of disease progression, is inadequate, which highlights the need for better prognostic indicators for NPC.

Cancer-related inflammation has been identified as a crucial host-related factor that may negatively affect the overall survival of patients with cancer, because inflammatory responses can trigger changes in the immune system, which lead to inhibiting effective immune responses. In addition, inflammatory responses can alter tumor cell biology [6,7]. It is now increasingly recognized that the systemic inflammatory response plays an important role in carcinogenesis and tumor progression [8,9,10]. Many studies indicated that the systemic inflammatory response was associated with a poor prognosis in patients with cancer. The main components of inflammation-based prognostic scores, including the Glasgow Prognostic Score (GPS), modified Glasgow Prognostic Score (mGPS), C-reactive protein/albumin (CRP/Alb) ratio, platelet–lymphocyte ratio (PLR), and neutrophil–lymphocyte ratio (NLR), have been reported to have prognostic significance in many types of cancers [11,12,13,14]. However, most of these studies only evaluated one or two biomarkers, without considering others. In this retrospective study, therefore, we proposed a novel inflammation-based stage, named I stage, which is a combination of inflammation-based independent prognostic indexes for predicting the prognoses of patients with NPC.

## 2. Results

### 2.1. Patient Characteristics

The clinicopathological and laboratory characteristics of 409 NPC patients, and their association with overall survival (OS), are summarized in Table 1. Among these patients, 288 (70.4%) were males and 121 (29.6%) were females, with a median age of 45 years (range 18–77). In total, there were 77 (18.8%) early stage (I–II) NPC patients and 332 (81.2%) advanced stage (III–IV) patients in the cohort. Seventy-four (18.1%) patients received radiotherapy treatment, while there were 335 (81.9%) patients using chemoradiotherapy treatments. The median OS was 53.7 months for the entire cohort of patients, with a five-year overall survival of 79.7%.

### 2.2. Prognostic Value of Inflammation-Based Prognostic Scores in Nasopharyngeal Carcinoma (NPC)

**Figure 1:**
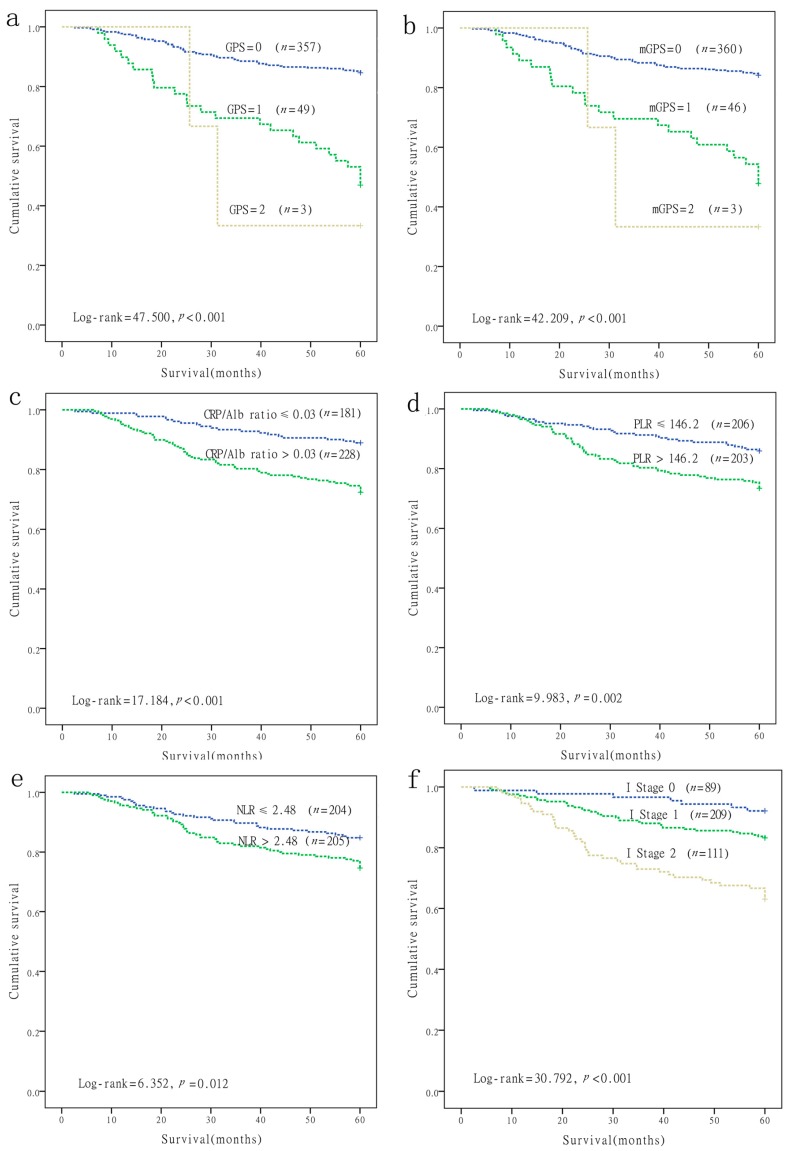
Kaplan-Meier survival curves of nasopharyngeal carcinoma (NPC) patients. (**a**) Overall survival of patients with GPS (*p* < 0.001); (**b**) Overall survival of patients with mGPS (*p* < 0.001); (**c**) Overall survival of patients with CRP/Alb ratio (*p* < 0.001); (**d**) Overall survival of patients with PLR (*p* = 0.002); (**e**) Overall survival of patients with NLR (*p* = 0.012); (**f**) Overall survival of patients with I stage (*p* < 0.001).

Patient survival curves were constructed via the Kaplan-Meier method, and were compared using the log-rank test. As shown in Figure 1 and Table 1, the significant prognostic indexes identified by univariate analysis included gender (*p* = 0.004), age (*p* = 0.002), tumor stage (*p* = 0.006), node stage (*p* < 0.001), TNM stage (*p* = 0.001), distant metastases (*p* < 0.001), treatment (*p* = 0.047), GPS (*p* ≤ 0.001), mGPS (*p* < 0.001), CRP/Alb ratio (*p* < 0.001), PLR (*p* = 0.002), and NLR (*p* = 0.012), and they were significantly predictive of five-year OS. The Cox proportional hazards model for multivariate analyses revealed that patients with a CRP/Alb ratio >0.03 had worse OS than patients with a CRP/Alb ratio ≤0.03 (Hazard ratio (HR) = 2.093; 95% Confidence interval (CI): 1.222–3.587; *p* = 0.007), and PLR > 146.2 had worse OS than patients with a PLR ≤ 146.2 (HR: 2.003; 95% CI: 1.177–3.410; *p* = 0.010), whereas GPS, mGPS, and NLR were not independent prognostic factors for OS in NPC (Table 2).

### 2.3. A Novel Inflammation-Based Stage (I Stage) Was Constructed by Combining Independent Risk Factors

According to the results of the multivariate analyses, the CRP/Alb ratio and PLR were independent prognostic factors in patients with NPC. Thus, the I stage was calculated as follows: Patients with both high levels of CRP/Alb ratio (>0.03) and an elevated PLR (>146.2) were assigned a score of 2, and patients with either or neither were assigned a score of 1 or 0, respectively. Of the 409 patients, 89 (21.8%) were allocated as I stage 0, 209 (51.1%) were allocated as I stage 1, and 111 (27.1%) were allocated as I stage 2, respectively. The five-year OS rates for patients with stages I0, I1, and I2 were 92.1% ± 2.9%, 83.3% ± 2.6%, and 63.1% ± 4.6%, respectively (*p* < 0.001, Figure 1f).

### 2.4. The Relationship between I Stage and Clinicopathological Characteristics

The relationship between the I stage and clinicopathological characteristics in NPC patients is shown in Table 3. There were no differences in the distribution of gender (*p* = 0.402), age (*p* = 0.487), and treatment (*p* = 0.831) in the I stage. However, the I stage was associated with the tumor stage (*p* = 0.038), node stage (*p* < 0.001), TNM stage (*p* = 0.014), and distant metastases (*p* < 0.001).

### 2.5. Comparison of the AUCs for the Inflammation-Based Prognostic Scores

**Figure 2:**
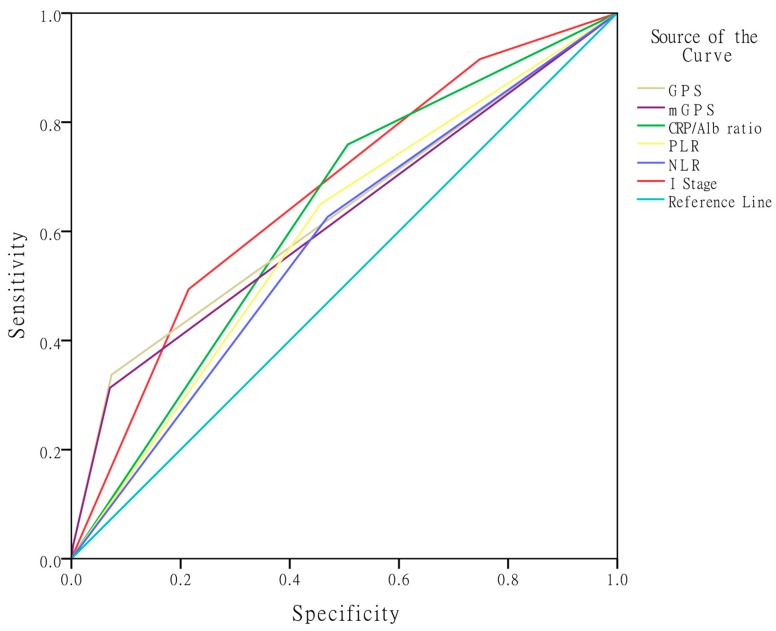
Comparison of the discriminatory ability of the I stage with GPS, mGPS, CRP/Alb ratio, PLR, and NLR.

The area under ROC curve (AUC) values were used to assess the discrimination ability of the I stage compared with the other inflammation-based prognostic indexes: GPS, mGPS, CRP/Alb ratio, PLR, and NLR (Table 4, Figure 2). The discrimination ability of the I stage, as assessed by AUC, was 0.670 (95% CI: 0.606–0.735; *p* < 0.001), which was higher than that of the other scores (GPS: 0.632, mGPS: 0.622, CRP/Alb ratio: 0.626, PLR: 0.597, and NLR: 0.579).

## 3. Discussion

In the present study, we proposed a novel inflammation-based prognostic system, named the I stage, which is a combination of independent prognostic factors (CRP/Alb ratio and PLR) in patients with NPC. We found that the I stage was a more accurate and useful prognostic score for the prediction of OS in patients with NPC. Additionally, we revealed that the I stage was associated with the tumor stage, node stage, TNM stage, and distant metastases.

Several hematological biomarkers have shown prognostic values in cancers. In particular, inflammation-based prognostic systems have been well studied. Cancer-associated inflammation was a key determinant of cancer initiation, progression, metastasis, and survival [15]. The systemic inflammation-based prognostic factors mainly include GPS, mGPS, CRP/Alb ratio, PLR, and NLR, which were associated with prognosis in various types of cancers [7,14,16,17,18,19,20,21,22]. Previous reports demonstrated that the systemic inflammatory response plays an important role in carcinogenesis and tumor progression [15,23]. Probable mechanisms were that inflammation was associated with malnutrition, immune dysfunction, up-regulation of growth factors, and angiogenesis [24,25]. Systemic inflammatory changes could promote the release of neutrophils, and some studies have reported the important role of elevated neutrophils and lymphocytopenia in inducing tumor angiogenesis [26]. Inflammatory responses can trigger changes in the immune system, which can inhibit effective immune responses by suppressing the cytolytic activity of lymphocytes [27]. Moreover, inflammatory responses can activate cytokine production and recruit regulatory T lymphocytes, which play an important role in tumor angiogenesis, invasion, and metastasis [28]. Some research has validated that elevated CRP levels have an impact on the growth and progression of cancers, including NPC [29]. GPS was derived from the acute-phase proteins C-reactive protein and albumin, which were more sensitive and reliable markers that reflect the systemic inflammatory response in cancer patients. Until now, GPS has been shown to be a favorable predictor of survival in patients with various cancers [30,31,32,33]. Recently, more researchers are focusing on investigating the modified GPS, named mGPS, which also is a combination of C-reactive protein and albumin [34]. Some studies have reported mGPS to be a powerful prognostic factor in many types of tumors [35,36,37]. The CRP/Alb ratio was primarily found in the outcome of acute exacerbations of chronic disease by Fairclough [17]. In further research on the CRP/Alb ratio for predicting the overall survival of patients with cancer, available research indicated that the CRP/Alb ratio was an efficacious prognostic factor in hepatocellular carcinoma, esophageal squamous cell carcinoma and NPC [14,38,39]. In addition, other prognostic scores (PLR, NLR) have also been actively studied in patients with NPC [40].

In our study, we investigated the prognostic values of systemic inflammatory parameters in NPC. This was not the first study to support the prognostic validity of the systemic inflammatory response in cancer. The results of the univariate analysis showed that the GPS, mGPS, CRP/Alb ratio, PLR, and NLR were predicting factors in NPC. These were similar to the above-mentioned results. However, the Cox regression analysis showed that only PLR and the CRP/Alb ratio were independent prognostic factors of overall survival (OS) in patients with NPC, whereas GPS, mGPS, and NLR were not. It is likely that most studies only focused on partial inflammation-based prognostic factors in cancer, especially in NPC, and there have been no studies assessing all five systemic inflammatory parameters to investigate the overall survival of patients with NPC. Strikingly, we found that the CRP/Alb ratio and PLR were the only inflammation-based scores with independent prognostic predictions on OS.

According to the results of the multivariate analyses, we proposed a novel inflammation-based stage (the I stage) combination of the CRP/Alb ratio and PLR; the I stage was divided into three groups: I0, I1 and I2. Then, the new I stage was applied to analyze the five-year overall survival of patients with NPC; our results showed that the five-year OS in patients with I0 was higher than that of I1 and I2, and the I2 group had the worst five-year survival rate.

Interestingly, when the correlations between the I stage and clinicopathologic characteristics were analyzed, a significant association was found between the I stage and TNM stage. Presently, the TNM staging system is commonly used to assess the prognosis of cancer. It may well be that the influence of the I stage on the subgroup with the TNM stage is important in the understanding of its role in patients with NPC.

In addition, we compared the I stage with the other inflammation-based prognostic factors (GPS, mGPS, CRP/Alb ratio, PLR, and NLR) using AUC values to assess the discriminatory ability. Our results showed that the AUC value of I stage was higher than that of the others. Thus, this result presented a more convincing conclusion that the I stage is a novel and more accurate predictive factor in patients with NPC; however, it still needs to be validated on a large-scale in NPC groups.

Besides these strengths, we recognize the study’s limitations. First, our study was a retrospective study; Second, the sample size was small, and we only carried out an analysis of 409 patients. In addition, this study was examined in a single center. So, the following research should be done on a large-scale and using multicenter prospective validation of NPC groups; Third, we were just concerned with the impact of systemic inflammation–based prognostic factors on the prognosis of patients with NPC, and we did not compare the novel inflammation-based prognostic system with other traditional prognostic factors, such as Epstein-Barr Virus DNA or lactate dehydrogenase (LDH) [41,42,43]. Thus, whether the I stage is more useful in predicting OS in patients with NPC as compared to other traditional prognostic factors remains to be ascertained.

## 4. Materials and Methods

We enrolled 409 patients, newly diagnosed with nasopharyngeal carcinoma, from Sun Yat-sen University Cancer Center (SYSUCC, Guangzhou, China), between January 2009 and January 2010. Peripheral blood samples were obtained from NPC patients before treatment, C-reactive protein (CRP) levels, albumin levels and complete blood cell counts at diagnosis were evaluated. The inclusion criteria for this study were as follows: (1) Histological confirmation of NPC and no previous anticancer treatment; and (2) available clinical information and laboratory data. Patients were excluded if there were detectable inflammatory disease, hematologic disorder, or another type of malignancy. The tumor stage was classified according to the Union for International Cancer Control/American Joint Committee on Cancer (UICC/AJCC) TNM classification system (6th edition, 2002). Our study was approved by the Sun Yat-sen University Cancer Center research ethics committee (Identification code: GZR2016-066; Date: 23 February 2016). The last follow-up date was 30 January 2015. Overall survival (OS) was calculated from the date of diagnosis to the date of death or last follow-up.

Use of GPS was demonstrated by previous studies [44]. Briefly, patients with both elevated C-reactive protein (>10 mg/L) and hypoalbuminemia (<35 g/L) were assigned a score of 2. Patients with either or neither were assigned a score of 1 or 0, respectively. mGPS, which was modified GPS, was constructed as follows: patients who had both elevated CRP (>10 mg/L) and decreased albumin levels (<35 g/L) were allocated a score of 2; patients with only elevated CRP (>10 mg/L) were allocated a score of 1, and those with a normal CRP were allocated a score of 0 [16]. The CRP/Alb ratio was calculated by dividing the serum CRP level by the serum albumin level [17]. Platelet-to-lymphocyte ratio (PLR) was defined as the absolute platelets divided by the absolute lymphocyte count, and neutrophil-to-lymphocyte ratio (NLR) was calculated as the neutrophil count divided by the lymphocyte count [45]. CRP and albumin levels were measured by a Hitachi 7600 automated chemistry analyzer (Hitachi Co., Tokyo, Japan) and blood cell count by a Sysmex XE-5000 System (Sysmex Co., Kobe, Japan). Descriptive statistics of patient characteristics were presented as mean and 95% confidence intervals (CIs). Comparisons between groups were performed using the *χ*^2^ test for categorical variables. Patients were separately divided into two groups with high or low levels using the cut-off value, as below, according to the median values, CRP/Alb ratio (≤0.03/>0.03), PLR (≤0.03/>0.03), NLR (≤0.03/>0.03). Survival analysis and curves were performed according to the Kaplan–Meier method and compared using the log-rank test. A Cox proportional-hazard model for multivariable analysis was applied for the inflammation-based prognostic scores that proved to be significant in the univariate analysis. The novel I stage was constructed using independent risk factors according the results of multivariate analyses. The discriminatory ability of the factors to predict OS were assessed using the area under the curve (AUC). Statistical analyses were performed using IBM SPSS 19.0 software (IBM Corporation, Armonk, NY, USA). All of the tests were two-sided, and *p* < 0.05 was considered statistically significant.

## 5. Conclusions

In conclusion, we proposed a novel inflammation-based stage combination of independent inflammation-related risk factors (CRP/Alb ratio and PLR). The I stage was superior to other inflammation-based prognostic indexes in terms of prognostic ability. Additionally, it was associated with tumor stage, node stage, TNM stage, and distant metastases. We believe that the I stage is a novel, more accurate, and useful predictive factor in patients with NPC. It is of significance to help clinicians identify high-risk patients and to enable post-treatment targeted rational therapy. In addition, the I stage is more objectively determined, and would be a simple, optimal, and inexpensive prognostic indicator in patients with NPC.

## Figures and Tables

**Table 1:** Univariate analyses of clinical and laboratory characteristics of survival time (S.T.) and the five-year overall survival (OS).

Patient Characteristics	No. of Patients (%)	S.T. (Month) Mean (95%, CI)	Five-year OS (%) Mean ± SD	*p*-Value
Gender				0.004
Male	288 (70.4%)	52.4 (50.6–54.2)	76.0 ± 2.5	
Female	121 (29.6%)	56.9 (55.1–58.7)	88.4 ± 2.9	
Age (years)				0.002
≤45	216 (52.8%)	55.0 (53.2–56.8)	85.6 ± 2.4	
>45	193 (47.2%)	52.3 (50.1–54.5)	73.1 ± 3.2	
Tumor stage				0.006
T1	38 (9.3%)	57.4 (55.1–59.8)	86.8 ± 5.5	
T2	109 (26.7%)	55.8 (53.3–58.2)	88.1 ± 3.1	
T3	161 (39.4%)	53.1 (50.7–55.5)	78.9 ± 3.2	
T4	101 (24.7%)	51.2 (48.1–54.3)	69.3 ± 4.6	
Node stage				<0.001
N0	75 (18.3%)	55.5 (52.7–58.3)	82.7 ± 4.4	
N1	140 (34.2%)	57.5 (56.1–58.9)	87.9 ± 2.8	
N2	149 (36.4%)	52.6 (50.0–55.2)	79.2 ± 3.3	
N3	45 (11.0%)	42.9 (37.0–48.8)	51.1 ± 7.5	
TNM stage				0.001
I–II	77 (18.8%)	58.9 (57.5–60.3)	93.5 ± 2.8	
III–IV	332 (81.2%)	52.5 (50.9–54.2)	76.5 ± 2.3	
Distant metastases				<0.001
Yes	64 (15.6%)	34.2 (29.9–38.5)	18.8 ± 4.9	
No	345 (84.4%)	57.4 (56.3–58.5)	91.0 ± 1.5	
Treatment				0.047
Radiotherapy	74 (18.1%)	57.8 (55.8–59.8)	87.8 ± 3.8	
Chemoradiotherapy	335 (81.9%)	52.9 (51.2–54.5)	77.9 ± 2.3	
GPS				<0.001
0	357 (87.3%)	55.0 (53.7–56.4)	84.6 ± 1.9	
1	49 (12.0%)	45.2 (39.7–50.7)	46.9 ± 7.1	
2	3 (0.7%)	39.0 (22.0–56.0)	33.3 ± 27.2	
mGPS				<0.001
0	360 (88.0%)	55.0 (53.5–56.3)	84.2 ± 1.9	
1	46 (11.3%)	45.4 (39.7–51.2)	47.8 ± 7.4	
2	3 (0.7%)	39.0 (22.0–56.0)	33.3 ± 27.2	
CRP/Alb ratio				<0.001
≤0.03	181 (44.3%)	56.8 (55.2–58.3)	89.0 ± 2.3	
>0.03	228 (55.7%)	51.3 (49.2–53.5)	72.4 ± 3.0	
PLR				0.002
≤146.2	206 (50.4%)	55.8 (54.1–57.5)	85.9 ± 2.4	
>146.2	203 (49.6%)	51.6 (49.4–53.9)	73.4 ± 3.1	
NLR				0.012
≤2.48	204 (49.9%)	55.2 (53.4–57.0)	84.8 ± 2.5	
>2.48	205 (50.1%)	52.3 (50.1–54.4)	74.6 ± 3.0	

CI: Confidence interval; TNM: Tumor-node-metastasis; GPS: Glasgow Prognostic Score; mGPS: Modified GPS; CRP/Alb ratio: C-reactive protein/Albumin ratio; PLR: Platelet-lymphocyte ratio; NLR: Neutrophil-lymphocyte ratio; SD: Standard deviation.

**Table 2:** Multivariate Cox regression analyses of the inflammation-based prognostic scores of nasopharyngeal carcinoma (NPC) patients.

Prognostic Factor	Coefficient	SE	*p*-Value	RR	95.0% CI for RR
GPS	1.404	0.727	0.053	4.070	0.980–16.908
mGPS	−0.382	0.741	0.606	0.682	0.160–2.9149
CRP/Alb ratio	0.739	0.275	0.007	2.093	1.222–3.587
PLR	0.695	0.271	0.010	2.003	1.177–3.410
NLR	0.140	0.266	0.598	1.150	0.683–1.938

SE: Standard error; RR: Risk ratio; CI: Confidence interval.

**Table 3:** Relationship between the I stage and clinicopathologic characteristics.

Patient Characteristics	I Stage 0	I Stage 1	I Stage 2	*p*-Value
	*n* = (89), No. (%)	*n* = (209), No. (%)	*n* = (111), No. (%)	
Gender				0.402
Male	66 (74.2%)	141 (67.5%)	81 (73.0%)	
Female	23 (25.8%)	68 (32.5%)	30 (27.0%)	
Age (years)				0.487
≤45	52 (58.4%)	107 (51.2%)	57 (51.4%)	
>45	37 (41.6%)	102 (48.8%)	54 (48.6%)	
Tumor stage				0.038
T1	4 (4.5%)	28 (13.4%)	6 (5.4%)	
T2	31 (34.8%)	53 (25.4%)	25 (22.5%)	
T3	33 (37.1%)	82 (39.2%)	46 (41.4%)	
T4	21 (23.6%)	46 (22.0%)	34 (30.6%)	
Node stage				<0.001
N0	15 (16.9%)	43 (20.6%)	17 (15.3%)	
N1	40 (44.9%)	76 (36.4%)	24 (21.6%)	
N2	31 (34.8%)	74 (35.4%)	44 (39.6%)	
N3	3 (3.4%)	16 (7.7%)	26 (23.4%)	
TNM stage				0.014
I–II	22 (24.7%)	44 (21.1%)	11 (9.9%)	
III–IV	67 (75.3%)	165 (78.9%)	100 (90.1%)	
Distant metastases				<0.001
Yes	5 (5.6%)	29 (13.9%)	30 (27.0%)	
No	84 (94.4%)	180 (86.1%)	81 (73.0%)	
Treatment				0.831
Radiotherapy	17 (19.1%)	39 (18.7%)	18 (16.2%)	
Chemoradiotherapy	72 (80.9%)	170 (81.3%)	93 (83.8%)	

**Table 4:** Comparison of the areas under the curves (AUCs) for the inflammation-based prognostic scores.

Scores	AUC	95% CI	*p*-Value
GPS	0.632	(0.559–0.706)	<0.001
mGPS	0.622	(0.548–0.695)	0.001
CRP/Alb ratio (categorical)	0.626	(0.562–0.691)	<0.001
PLR (categorical)	0.597	(0.529–0.664)	0.006
NLR (categorical)	0.579	(0.510–0.647)	0.027
I Stage (categorical)	0.670	(0.606–0.735)	<0.001
